# Supplementary material for: Patterns of Intron Gain and Loss in Fungi
Source: PLoS Biol. 2004 Nov 30;2(12):e422. doi: 10.1371/journal.pbio.0020422 (PMC532390; doi:10.1371/journal.pbio.0020422)
Supplement: Table S1 — Also available at http://genes.mit.edu/NielsenEtAl/. (4.3 MB ZIP). [file pbio.0020422.st001.zip › NielsenEtAl/html/758.html]

AN5812.1.NCU06732.1.MG09481.1.FG10188.1


```
 CLUSTAL W (1.82) Multiple Sequence Alignments - Introns Inserted


Sequence 1: NCU06732.1	614 aa
Sequence 2: MG09481.1	613 aa
Sequence 3: FG10188.1	639 aa
Sequence 4: AN5812.1	639 aa
Alignment Length: 654 aa
Number Identitical Residues: 281 aa
Alignment Score (without introns) 15233


MG09481.1 	---------------------~--------------MAARDPSTASNYDAWKTKHTTANL
NCU06732.1	---------------------~-------------MAPVRDPNTLSNYDAWRTRHTTANL
FG10188.1 	MSWFSSLKKPSILRTCQVTIT~HNLRRTFATVNPNMTANRDPSTLSNYSAWRTRHTTANF
AN5812.1  	-------MTTTTTTTTTTTLP2FNLYG--RSMATLMNPVRDPNTLSNYNNWLCTHTTANF
          	        ..:   :   : .  .      :  .   . ***.* ***. *   *****:

MG09481.1 	RIDFDDKCLRGSVVLELESRTAKESKEIVLDSSYVSVESIKLNDVQTK-WEIKERNGPMG
NCU06732.1	KIDFTAKCLRGSVILELESQTDKASKEIVLDSSYVTVNSIKLNSAPSL-WETKARTEPNG
FG10188.1 	KIDFDQKALKGSVVLQLESQTDKESKEIILDTRYVDISSISIDSKESK-WELKSFNAPLG
AN5812.1  	EIFFEEKKLVGNVVHKLRSITNAETDEIILDSHHVDIRNVQVAGLPVKAWELLPPLGPYG
          	.* *  * * *.*: :*.* *   :.**:**: :* : .:.: .    :**      * *

MG09481.1 	SPLHISVPEGADSGEVVRLE~MAVKTTPQCTALQWLTPAQTSNKKAPFMF~SQAQACHAR
NCU06732.1	SPVHIAVPEGAAKGEVVKVE~IELATTDKCTALQWLTPAQTSDKAAPFMF~SQCQAIHAR
FG10188.1 	APLHIAVPDGVAKGELIDLA~IDLQTTSKCTALQWLTPAQTSNKKHPYMF~SQCQAINAR
AN5812.1  	TALKIKLENPVGLNEIIDVD0IAVQTTKECTALQWLTPAQTSNKKHPYMF1SQCQAIHAR
          	:.::* : : .  .*:: :  : : ** :*************:*  *:** **.** :**

MG09481.1 	SLFPCQDTPDVKSTYSFNITSPHVVVASGVANDGDKAEAD-GGDKVYKYEQNVPIPSYLF
NCU06732.1	SLFPCQDTPDVKSTYDFNITSPYVVVASGVPVP-DETKDL-GEEKLYKFQQKVPIPSYLF
FG10188.1 	SIFPCQDTPDVKSTFSFKLTSSLPVVASGVPVG-DHEATP-GKEKLYEFEQKVPIPSYLF
AN5812.1  	SIFPCQDTPDVKCTFDFNITSPLPVIASGLPVRSTSTVPQSGVKTLHRFHQKVPIPSYLF
          	*:**********.*:.*::**.  *:***:.  .      :* ..::.:.*:********

MG09481.1 	ALASG~DIAMAPIGPRSSVATGPDEVKECQWELEEDMGKFMDAAE~RLVFPYRWGEYNVL
NCU06732.1	ALSSG~EIASAPVGKRSCVCTGPNELKASQWELEGDMDKFLEAAE~KIVFPYRWGEYNVL
FG10188.1 	AVASG~DIATAPIGPRSIVATGPNELEGCKWELERDMEKFMEVAE~KLVFPYKWGAYNVL
AN5812.1  	ALASG2DIAEAAIGPRSVVATSPDKLEECKWELEADTERFIKTIE0EIIYPYAWGEYNVL
          	*::** :** *.:* ** *.*.*:::: .:**** *  :*:.. * .:::** ** ****

MG09481.1 	VLPPSFPYGG1MENPIYTFATPTIISG~DRQNTDVIAHELSHSWSGNLVTSCSWEHY~WL
NCU06732.1	VLPPSFPYGG1MENPIFTFATPTIISG~DKQNIDVIAHELAHSWSGNLVTSCSWEHF2WL
FG10188.1 	VLPPSFPYGG~MENPIYTFATPTIISG~DRQNVDVIAHELSHSWSGNLVSNASWEHF2WL
AN5812.1  	ILPPSFPYGG~MENPVFTFATPSIISK0DRENVDVIAHELAHSWSGNLVTSASWEHF~WL
          	:********* ****::*****:***  *::* *******:********:..****: **

MG09481.1 	NEGWTMYLERR~IIAAV~RG-PAYFDFSALLGWKHLE1DAIEEFGADHKFTQLCINHKGI
NCU06732.1	NEGWTMYLERR~ILASI~HGGDAHFDFSAIRGWKALE1EAIKEYGEDHEFTKLCISHKGI
FG10188.1 	NEGWTMYLERR~IQAAI~HG-DAEFDFSAIIGWKDLE1NAVELFGKDHEYTKLIIKHEGV
AN5812.1  	NEGWTVYLERR0ILASL2HG-EKYRHFSAIIGWKALR~DSVEHYSHDHEFTKLVPNLKGE
          	*****:***** * *:: :*     .***: *** *. :::: :. **::*:*  . :* 

MG09481.1 	DPDDAFSTVPYEKGFHMVYYLDRLVGRKNFDKFIPY~YFTKWANKSLDSYEFKDTFLEFF
NCU06732.1	DPDDAFSTVPYEKGFHFVWSLDRLVGRENFDKFIPY~YFGKWSNKSLDSYEFKDTFLEFF
FG10188.1 	DPEDVYSTVAYEKGFHFLYYLEGVVGRENFDKFIPF~YFTKWSEKSLDSFEFKQTFLDFF
AN5812.1  	DPDDAFSTIPYEKGFNFLFHLENLVGKEKFDRFIPH0YFTTFKGKSLDSYDFKATLLDFF
          	**:*.:**:.*****:::: *: :**:::**:***. ** .:  *****::** *:*:**

MG09481.1 	DKPEYADLKDKIAGIDWEGRFYTPGLPPKPEFDTSLVDVCYALADKWK---K0G~DYTPS
NCU06732.1	SAPEYSDLKDKIASIDWEGRFHSTGLPPKPEFDTSLADVCYELAEKWK---S~K~DFTPS
FG10188.1 	NNLGNKEVAKKITEINWEEKFYTPGLPPKPEFDTTLANQCYDLANKWK---D~A0KFEPN
AN5812.1  	KS--DAEASRLLQELDWDSWFYKPGLPPKPEFDTSLADVVYELAGKWRSLPE~S~PFQPQ
          	.     :    :  ::*:  *:..**********:*.:  * ** **:: ..    : *.

MG09481.1 	SKDVDGWTGNQKLVFLGSVQDFEQPLSAEQA~KQLGNAYDLIETKNVELKTAYYLIALRA
NCU06732.1	PSDVASWTGNQVLVFLNAVQDFEEPLTVEQS0QALGKAYGLSESKNAELKAAYYHIAMRS
FG10188.1 	ANDLEEFTANQKLVFLGEVQQSGD-LTADRV~QLMGKTYDFMSSKNVEILSAYYQIALQA
AN5812.1  	PSDIQGLTANQIVVFLEQILLFERPLTAELS~KLMGEVYGLTGSENIEVANLYLQVGLKA
          	..*:   *.** :***  :     .*:.:   : :*:.*.:  ::* *:   *  :.:::

MG09481.1 	QDSTAYQGVADLLGQVGRMKFVRPL~FRALNKVDRPLALTTFEKNKDFYHPICRAMAEKD
NCU06732.1	KDASAYQGVADLLGEVGRMKFVRPL~FRGLNKVDRELALKTFEKNREFYHPICRQMVEKD
FG10188.1 	KDSAIYQAAADLLGTVGRMKYVRPL~FRALNKVDRQLALDTFEKNKDFYHPICKGMVEKD
AN5812.1  	ADKSVIGPTTDLLGRIGRMKFVRPL2YRALQKVDRQVAIDTFEKHKDFYHPICRGMVEKD
          	 * :    .:**** :****:**** :*.*:**** :*: ****:::******: *.***

MG09481.1 	LGLSDDAKKE
NCU06732.1	LGVSEAANSS
FG10188.1 	LGL-------
AN5812.1  	LFGKKDA---
          	*  .. :
```
